# Recording Lifetime Behavior and Movement in an Invertebrate Model

**DOI:** 10.1371/journal.pone.0018151

**Published:** 2011-04-12

**Authors:** Sige Zou, Pablo Liedo, Leopoldo Altamirano-Robles, Janeth Cruz-Enriquez, Amy Morice, Donald K. Ingram, Kevin Kaub, Nikos Papadopoulos, James R. Carey

**Affiliations:** 1 Laboratory of Experimental Gerontology, National Institute on Aging, Baltimore, Maryland, United States of America; 2 Departamento de Entomología, El Colegio de la Frontera Sur, Tapachula, Chiapas, Mexico; 3 Laboratorio de Visión por Computadora, Instituto Nacional de Astrofísica, Óptica y Electrónica, Tonantzintla, Puebla, Mexico; 4 Department of Entomology, University of California Davis, Davis, California, United States of America; 5 Nutritional Neuroscience and Aging Laboratory, Pennington Biomedical Research Center, Louisiana State University, Baton Rouge, Louisiana, United States of America; 6 Department of Agriculture Crop Production and Rural Environment, University of Thessaly, Magnisias, Greece; 7 Center for the Economics and Demography of Aging, University of California, Berkeley, Berkeley, California, United States of America; Buck Institute for Age Research, United States of America

## Abstract

Characterization of lifetime behavioral changes is essential for understanding aging and aging-related diseases. However, such studies are scarce partly due to the lack of efficient tools. Here we describe and provide proof of concept for a stereo vision system that classifies and sequentially records at an extremely fine scale six different behaviors (resting, micro-movement, walking, flying, feeding and drinking) and the within-cage (3D) location of individual tephritid fruit flies by time-of-day throughout their lives. Using flies fed on two different diets, full sugar-yeast and sugar-only diets, we report for the first time their behavioral changes throughout their lives at a high resolution. We have found that the daily activity peaks at the age of 15–20 days and then gradually declines with age for flies on both diets. However, the overall daily activity is higher for flies on sugar-only diet than those on the full diet. Flies on sugar-only diet show a stronger diurnal localization pattern with higher preference to staying on the top of the cage during the period of light-off when compared to flies on the full diet. Clustering analyses of age-specific behavior patterns reveal three distinct young, middle-aged and old clusters for flies on each of the two diets. The middle-aged groups for flies on sugar-only diet consist of much younger age groups when compared to flies on full diet. This technology provides research opportunities for using a behavioral informatics approach for understanding different ways in which behavior, movement, and aging in model organisms are mutually affecting.

## Introduction

Aging is associated with numerous behavioral changes and a gradual decline of physiological function. Despite an extensive literature on insect behavior and movement including many studies involving *Drosophila *spp [1], [2], [3] and a rapidly growing interest in the role of behavior in aging research [2], there is a remarkable paucity of studies on these topics concerned with age-specific and lifetime patterns. This dearth stems partly from the relatively low interest in behavioral research in biogerontology until quite recently, and partly from an absence of an automated system for recording the details of daily and lifetime behavior and movement. The published systems used in fruit fly behavior research include the *Drosophila* Activity Monitoring System [4], [5] used in sleep research, several electronic video systems that use recorded trajectories to infer behavioral and/or movement patterns [6], [7], [8], [9] and a number of systems with specialized concepts for recording fly activity [10], [11], [12]. Herein we describe and provide proof of concept for a new behavioral monitor system (BMS) that is capable of continuously recording the lifetime behavior and movement of individual tephritid fruit flies, *Anastrepha ludens*. As with other tephritid fruit flies, *A. ludens* is used extensively in demographic studies of aging and aging interventions [13],[14]. Unlike other fly monitoring systems, the BMS records 3D location of the fly within the cage and the sequence of multiple behaviors at an extremely fine scale over a long period of time. The lifetime recording and behavioral classification are achieved by converting imaging files of a large size to much smaller sized text files in real time in our system. Using the BMS, we describe for the first time lifetime behavioral changes of flies at a high resolution, comparing two dietary conditions.

## Methods

### Fly husbandry

Pupae of tephritid fruit fly, *Anastrepha ludens*, were obtained from the “Moscafrut” mass rearing facility at Metapa, Chiapas, Mexico. Newly eclosed virgin females were randomly assigned to one of the nine cages in the BMS and maintained with water and food. Two BMS units were used to monitor lifetime behaviors of *A. ludens* females, each housing 4 females on each of the two diets—either full or sugar-only. The full diet contained sugar and yeast extract at 3∶1 ratio. Fresh food and water were provided to flies weekly. The study consisted of two replicate experiments yielding data on a total of 32 females yielding 16 individuals on each of the two diets.

### Behavioral monitoring system and assays

The BMS consists of a pair of cameras to acquire left and right video images in real time with visible and infrared lights, and a nine-cage tray to house individual flies. For the behavioral assay, the cameras and lighting were adjusted to capture clear left and right images of each cage in the center of video at the beginning of each experiment. After adjustment, the positions of food and water were recorded, and the fly was recognized as a white image in the dark background of the cage. The rate of sampling by cameras was 5 images per second with a video sampling time for experiments of 60 seconds. The behavioral recording was initiated once the positions of food and water, sex of the flies, date of birth, comments, and sampling time were loaded into the BMS. When fresh food and water were provided to flies weekly, the BMS was re-configured and re-adjusted in the same way as was done at the beginning of the experiments. Recording were initiated after the eclosion of flies and lasted throughout the lifetime for all flies except for five females on full diet and one female on sugar diet in the first replicate experiment which were terminated at day 134. The hardware design and software are available upon request.

### Criteria for behaviors

The classification of the behaviors (resting, micro-moving, walking, flying, feeding and drinking) was determined by applying the following rules to the 3-D path of the fly:

- Given two positions P(x,y,z) and P'(x',y',z') of the fly in two consecutive images, if 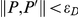, then the behavior is classified as resting. Otherwise it is micro-movement or other behaviors. Here 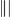 is the Euclidian distance, 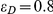 is the threshold in millimeter to identify the behavior resting.
- Given a consecutive point set 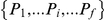, which is determined by the sampling time, if 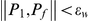 where 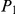, is the initial point and 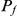 the final point of the set, then each point couple 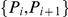 is marked as micro-moving. Otherwise, the behavior is marked as walking. 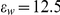 is the threshold in millimeter for walking.
- Given two consecutive points 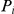 and 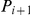, if 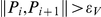, then the behavior is classified as flying. 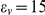
mm is the threshold for flying.
- Given a set of 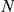 consecutive points marked as resting, if 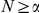 and the points are inside of a circle fixed at the food center with radius 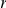 and 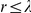, then the whole set is marked as feeding. 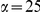 is the number of points needed to select the behavior feeding, 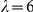 mm is the size of the circle around the food.
- Drinking behavior is marked in the similar way as that in (d) except that the circle is placed at water center.
- The fly is marked as dead if it is classified as resting for at least 5 hours.

### Data analysis

Behavior sequences and totals matching specific criteria (time, fly age, fly treatment) were extracted from daily log files using Python. This summary data were imported into R for normalization, processing, statistical analyses, and clustering analyses using TraMineR's sequence comparison functions or by Euclidean distances between averaged behavior levels. TraMineR is an R-package for mining and visualizing sequences of categorical data (http://mephisto.unige.ch/traminer/) [15] (Table S1, Table S2, Table S3).

For each behavior, the frequency or activity values in every hour of the day were summed up to generate hourly values in the function of age and hour of the day. Total distance traveled by a fly in every hour of the day was computed by adding all the activity values in that hour. The results were used to generate event history graphs depicting the patterns in the function of age or time of the day for each behavior using Microsoft Excel© program. To assess the age-related or circadian rhythm patterns, the average of hourly distance traveled by 32 flies on either full or sugar-only diet were computed over the age in days or time of the day in hours. The difference between age-related distance and activity patterns for flies on two diets was assessed by the Cox LogRank test. The difference between the peak activity levels or location preference for flies on the two diets was assessed by Student's *t*-test. A value of *p*<0.05 was considered as statistically significant. Bonferroni correction was applied to adjust the p values when multiple comparisons were involved in the statistical analysis.

## Results and Discussion

**Figure 1:**
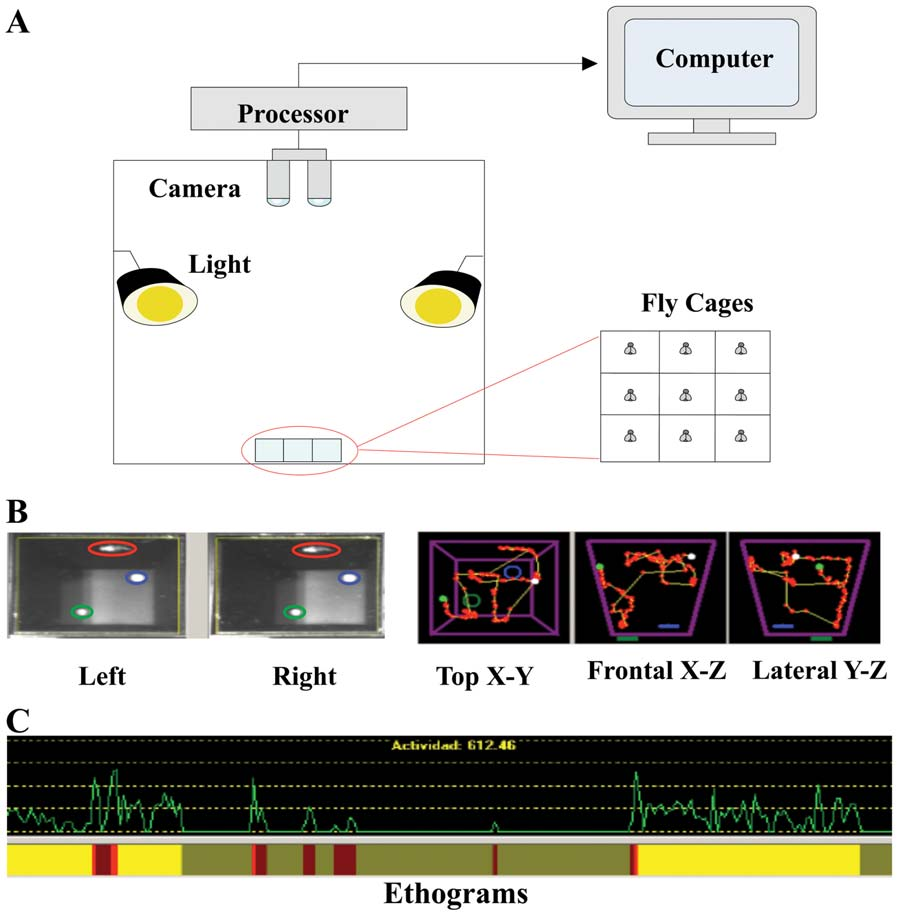
Overview of the behavior monitor system. (**A**) The hardware of the BMS consists of one 9-cage behavior arena, lighting, two cameras and a computer. Each cage has two fixed positions at the bottom to provide water and food (not depicted) to individually housed flies. (**B**) 3-D coordinate trajectories are generated by the software of the BMS from left and right video images taken by the two cameras. (**C**) The BMS software automates classification of six behaviors color coded in the ethogram based on the activity values and relative position of the fly to water and food sources using 3D trajectory data.

The BMS employs a pair of cameras and a computer to acquire stereo video images of the fly individually housed in a 9-cage tray (Fig. 1A). The left and right images captured by two cameras (Fig. 1B) are analyzed immediately after each sampling bout by the BMS software. The BMS stitches the constitutive frames to generate 3D trajectories of flies and then automates classification of six behaviors (resting, micro-movement, walking, flying, feeding, and drinking) for each frame by calculating changes of the 3D coordinates between two constitutive frames (see criteria in the Materials and Methods). In this study, each recording bout was 60 seconds/cage at 5 readings/sec, which resulted in 300 character sets with both the 3D coordinates and behavior readings per bout. Because the data processing in computer RAM required approximately one minute for every minute recording and there were 9 flies that were monitored sequentially, a new recording bout occurred once every 18–20 minutes for each fly (i.e. 2 or 3 one-minute bouts/hr).

To assess both accuracy and precision of the BMS, the visual recordings by technicians were compared to the output files from the BMS (Table S2). We found that the accuracy for the BMS to record the behaviors recognized by technicians was 98.4–100% for four behaviors, resting, micro-movements, walking and flying, indicating the reliability of the automated system. The accuracy for technicians to record the behaviors classified by the BMS was ∼100% for resting and walking, 90–100% for micro-movement and 70–100% for flying. The relatively lower accuracy to recognize micro-movement and flying by technicians was likely due to the limitation of human eyes to capture the subtleness of micro-movement and quickness of flying instead of false positives recorded by the BMS. Recordings for feeding and drinking were the least reliable since these were based on proximity to the food or water source for a certain period time, which did not necessarily mean that the fly was actually feeding or drinking.

**Figure 2:**
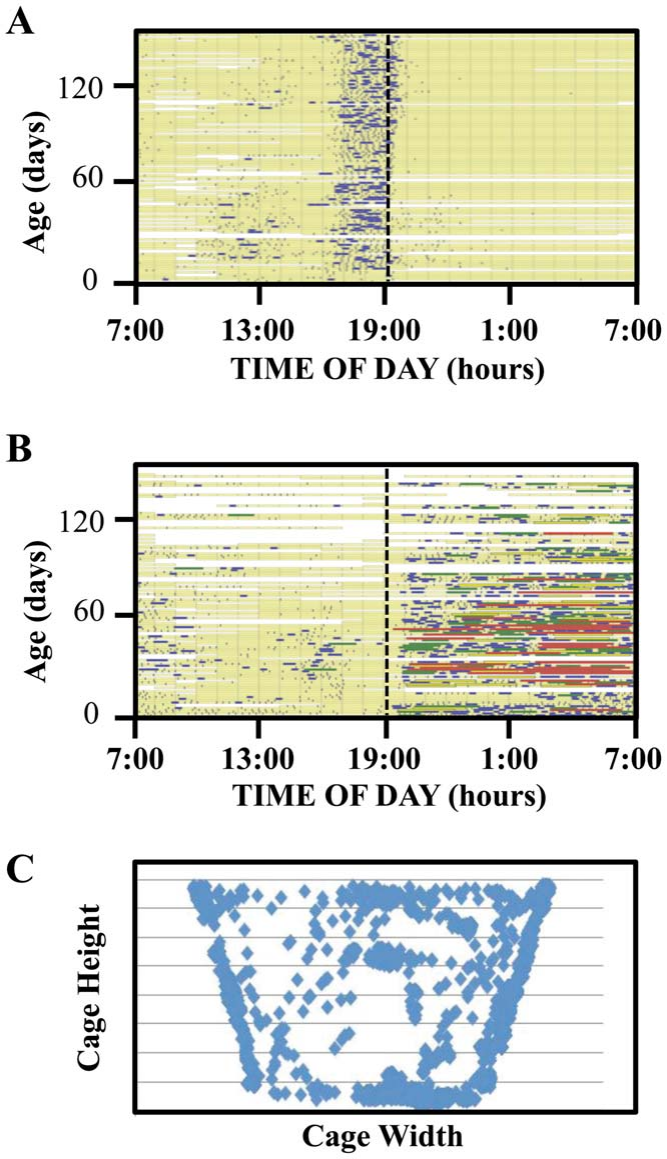
Behavior patterns of a single fly. (**A**) Event history chart for a representative individual fly on full diet depicting the patterns of walking by age and time-of-day (indexed at time 7∶00 for lights on; 19∶00 for lights off). Each day of the fly's life is shown as a horizontal line divided into 24 one-hour segments, each of which codes the 2 or 3 one-minute recordings per hour. Code: walking = blue; not walking = yellow; blank/white = no data. Lines containing the time-of-day information are ordered by age from bottom (youngest) to top (oldest). (**B**) Event-history chart for a representative individual fly on full diet showing the duration of rest (sleep-like) bouts (pale yellow  =  recorded data; blue, green, yellow, and red denote rest durations of <1, 1–2, 2–3, and >3 hrs, respectively). (**C**) Spatial graphic depicting the 24-hr within-cage movement of a 30 day-old female *A. ludens*. Only the front view is shown.

Examples of data output from the studies of *A. ludens* fed two diets, full or sugar-only, using the BMS are shown in Fig. 2 including event history charts by age and time-of-day showing walking patterns (Fig. 2A), rest bout duration (Fig. 2B) and within-cage location for a single fly over a 24-hour period (Fig. 2C). This indicates that the BMS can record and generate high-resolution behavioral patterns.

**Figure 3:**
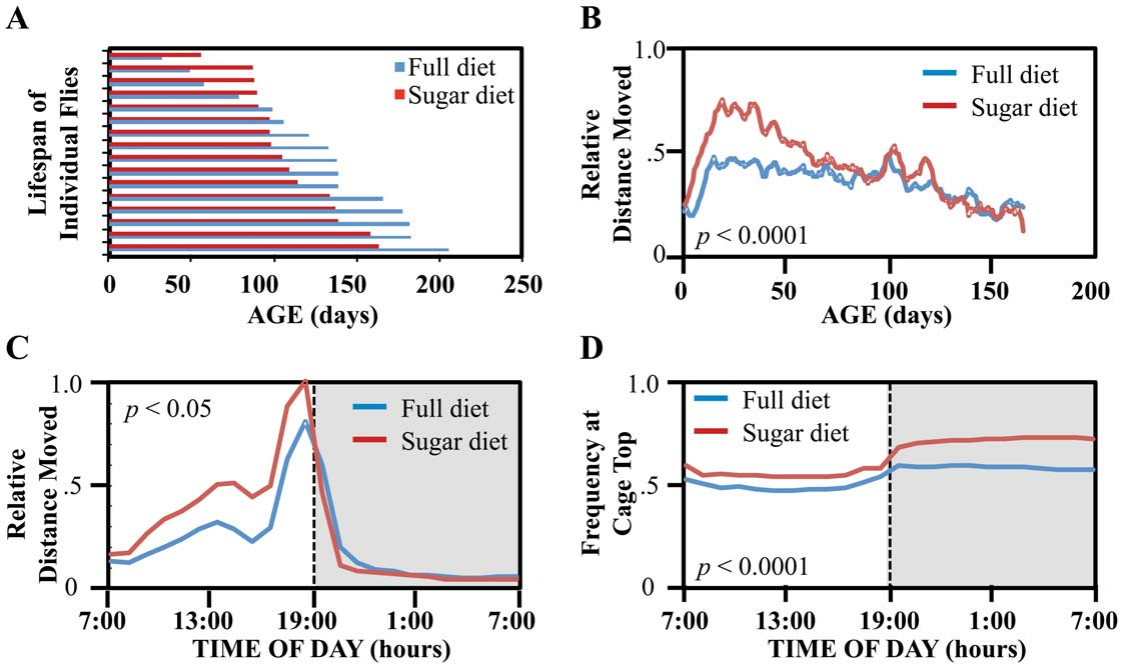
Age- and diet-specific behavior patterns of flies. (**A**) Lifspan of individual flies on full or sugar diet (red  =  full diet; blue  =  sugar-only diet; n = 16 females for each diet). (**B**) Normalized average age- and diet-specific schedules of daily distance moved for *A. ludens* females. (**C**) Normalized average diet-specific frequency distribution of distance moved by *A. ludens* females through a 24-hour period. Grey area indicates the lights off period. (**D**) Average diet-specific frequency of time spent at the top of the cage for *A. ludens* females through a 24-hour period. The p values indicating the difference in diet-specific changes are shown in each panel.

To assess the lifetime activity changes of flies, we calculated the distance that a fly travels every hour of a day through its life, assuming that the distance that a fly travels reflects how active it is. This analysis was performed for 32 female flies on full or sugar only diet in the first replicate study. As shown in Fig. 3A, the average lifespan of flies on full diet was longer than that on sugar only diet. By averaging daily or hourly total distance of individual flies, we generated age-related distance patterns (Fig. 3B) and circadian distance patterns (Fig. 3C) for these flies. The daily total distance gradually increased in the first 15 days, reached the peak at the age of approximately 20 days and then gradually declined with age for flies on both diets. During the first half of the lifespan, the daily distance was higher for flies on the sugar diet than those on the full diet with the peak distance levels differing by ∼60% between the two diets. The circadian distance patterns reveal that overall patterns were similar between flies on both diets with ∼90% of the daily distance traveled is during daytime (Fig. 3C). ∼50% of the distance traveled is in late afternoon from 3–7 pm and the distance peaks at 6 pm for flies on both diets. Consistent with the daily distance patterns, the hourly peak distance level for flies on sugar-only diet is ∼20% higher than that on full diet.

To examine the diet-specific changes in the location preference, we used the 3-D coordinates of 32 flies fed full or sugar-only diet to determine within-cage location preference for a fly with a 24-hour period. Fig. 3C shows the average diet-specific frequency of the time flies were located at the top of the cage by time-of-day within a 24-hour period over their lifetimes. Flies on the full diet spent 48–55% of the time at the top of the cage during the daytime (light-on period), and slightly more time (∼60%) during the night time (light-off period). On the other hand, flies on sugar only diet spent slightly more time (54–60%) at the top of the cage during the day time and much more time (69-74% of time) during the night time when compared to flies on full diet. This suggests that dietary conditions have significant impact on the diurnal location preference patterns.

**Figure 4:**
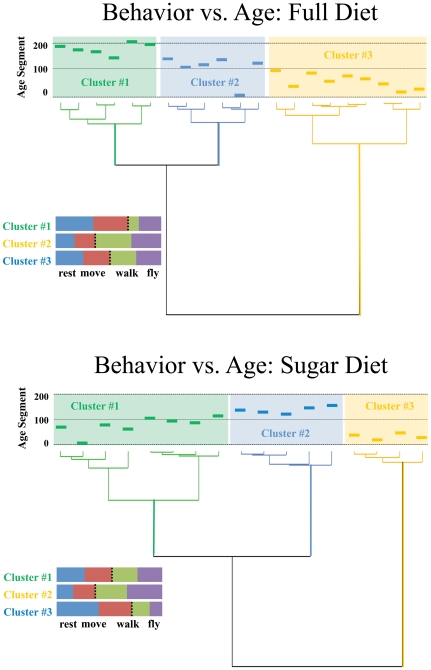
Dendrogram produced by software TraMineR showing the hierarchical clustering of age periods in *A. ludens* according to similarities in the lifetime behavior patterns. (A). Hietrarchical patterns for 16 females on full diet. The lengths of the branches connecting the internal nodes indicate degree of behavioral similarity and the leaf nodes represent elements of the behavior data (age intervals) used to construct the dendrograms. Each leaf in this graph represents the average behavior levels for the specified age range across all flies. These age classes are clustered by the Euclidean distance between their respective average behavior levels. The rectangles at the end of each ‘leaf’ (top of graphic) indicate the 10-day age intervals over which the behavior data was grouped and analyzed. The inset shows the relative differences in the frequency of each behavior that underlie the three clusters. Cluster #1 consists of oldest age groups, Cluster #2 for middle-aged groups and Cluster #3 for young age groups. Note the between-cluster differences in the behavioral distributions to the left and right of the dashed vertical lines; i.e. the rank-order between clusters for the proportion of time walking and flying is: Cluster #2>Cluster #3>Cluster #1. Conversely, the proportion of the time resting and micro-movement is: Cluster #1>Cluster #3>Cluster #2. (B). Hierarchical patterns for 16 females on sugar-only diet. Cluster #2 consists of oldest age groups, Cluster #1 for middle-aged groups and Cluster #3 for young age groups. The rank-order between clusters for the proportion of time walking and flying is: Cluster #2>Cluster #1>Cluster #3. Conversely, the proportion of the time resting and micro-movement is: Cluster #3>Cluster #1>Cluster #2. *p<0.05, **p<0.01 and ***p<0.001 indicates the similarity between age groups under a branch.

The ability of the BMS to generate a large amount of behavior data at a fine scale provides unique research opportunities for behavior informatics. Just as the study of genomics has become largely a digital science made possible by the advent of sequencing technology and powerful informatics software programs [16], the study of behavior using the BMS described here provided the opportunity to bring sequence data resources to bear on the *A. ludens* digital data. We used the genomics-inspired TraMineR software (http://mephisto.unige.ch/traminer/) to create two dendrograms for 32 females on full or sugar-only diet depicting the hierarchical clustering of different 10-day age groups according to age-specific behavior patterns (Fig. 4). The graphic presentation generated from 16 females on full diet reveals that: i) behavior patterns under 101 days are much different than at older ages as indicated by the long branches separating Cluster #3 (youngest ages) from Clusters #1 (oldest ages) and #2 (middle ages); ii) the behavioral patterns for age classes above 100 days are split into two unequal groups. Cluster #1 includes six oldest 10-day age groups between 141 and 210 days, while Cluster #2 consists of five 10-day age groups between 101 to 160 days and the single age group between 0 and 10 days; and iii) the behavioral ‘signature’ of the youngest flies (0-10 days) is most similar to middle-aged flies that are 121–140 days old as indicated by their inclusion in Cluster #2—a cluster containing all of the middle-aged flies. The dendrogram for 16 females on sugar-only diet also reveals three clusters of 10-day age groups. Behavior patterns for five oldest age groups above 120 days (Cluster #2) are much different than that for four young age groups between 11 and 50 days (Cluster #3). Cluster #1 includes seven middle-aged groups between 51 to 120 days and the youngest age group between 0 and 10 days. Similar to the patterns for flies on full diet, middle-aged groups are closer to oldest age groups than young age groups. However, the middle-aged groups of flies on sugar-only diet consist of much younger age groups (51–60 days) when compared to flies on full diet (101–110 days). Overall, our findings indicate that dietary nutrients play an important role in lifetime behavioral changes.

Recognizing the importance of high-resolution recording, several video tracking systems have been implemented to automatically record trajectory at a high resolution for individual or a group of animals including flies. Branson *et al*. have described a general purpose, high-throughput and automated system to quantify social and individual behaviors of *Drosophila* by automating analysis of trajectories of individual flies in a population (Branson *et al*. 2009). This system combines fly detection and identity assignment steps, which allows high-throughput screening and specifying new behavioral detectors without additional programming. However, this system tracks flies in a planar arena, which misses rich 3-D patterns of free living flies. Tower and his colleagues have developed a system capable of tracking movement of multiple *Drosophila *in 3-D [17]. They have recently described an improved 3-D system that can simultaneously tracking movement and gene expression of individual or multiple flies with GFP and DsRED fluorescent reporter transgenes [18], [19]. However, these 3-D *Drosophila* systems lack automated behavior detection features. In addition, none of the existing tracking systems has been demonstrated to actually have the capacity to automate recording and analyzing behaviors over the lifetime of an animal.

It is challenging to develop a lifetime tracking system and implement it in a typical biology laboratory. One challenge is that such system should have the capacity to run reliably the video recording and convert the imaging data of huge sizes to files of manageable size for storage in a standard lab computer for days, weeks and months. The other challenge is to automate characterization of different types of behaviors from the raw video recording data without additional programming. We have demonstrated here that the BMS as developed to date is a fully-automated video system with the capacity to generate lifetime high resolution behavioral data in 3-D for multiple individually housed flies without tagging and without additional programming in a typical biology laboratory. Data-intensive analyses such as the hierarchical clustering using the TraMineR software could be used to address questions concerned with hierarchical clustering of behaviors in a wide range of gerontological and demographic contexts including topics ranging from the oldest-old, male-female differences, and longevity mutants to dietary restriction, sleep patterns, and circadian rhythm. Further development of the software and hardware of the BMS should allow tracking group housed individual flies and other invertebrate flying species, such as *Drosophila*, house flies and mosquitoes.

### Conclusion

The BMS appears to be the first fully-automated video system capable of classifying the behaviors of individual flies in 3D space, of accurately recording their movement and within-cage location, and of generating fine-grained digital code sequences of behavioral states throughout their lives that can be analyzed using software derived from studies in computational biology and genomics. This technology provides researchers the opportunity to use a behavioral informatics approach for understanding the effects of behavior and movement on survival, aging and longevity in model organisms.

## Supporting Information

Table S1 **Template for validation of BMC system for Mexfly.**
(XLSX)

Table S2 **Summary statistics for validation studies of the BMS.**
(XLSX)

Table S3 **Metrics corresponding to leaves in the dendrogram depicted in Figure 3.**
(XLSX)
